# Effects of 5-Aza-2′-deoxycytidine on the methylation state and function of the WWOX gene in the HO-8910 ovarian cancer cell line

**DOI:** 10.3892/ol.2013.1438

**Published:** 2013-07-03

**Authors:** HONGCHAO YAN, NAN YU, JIANYE TONG

**Affiliations:** Department of Obstetrics and Gynecology, The Affiliated Hospital of Xuzhou Medical College, Xuzhou, Jiangsu 221002, P.R. China

**Keywords:** ovarian cancer, WWOX gene, 5-Aza-2′-deoxycytidine

## Abstract

The aim of this study was to explore the effects of 5-Aza-2′-deoxycytidine (5-Aza-CdR), a DNA methylation inhibitor, on the methylation state and function of the WWOX gene in the HO-8910 ovarian cancer cell line. The HO-8910 cells were divided into two groups, a control group and a 5-Aza-CdR-treated group. The methylation state of the WWOX gene was evaluated using a methylation-specific PCR assay. The effect of 5-Aza-CdR on the HO-8910 cells was analyzed using MTT and cell invasion assays, as well as flow cytometry. The animal models were established by intraperitoneal transplantation of the cells into nude mice. Following treatment with 5-Aza-CdR, a demethylation state was detected in the HO-8910 cells. WWOX protein expression was significantly higher in the 5-Aza-CdR-treated group compared with that in the control group. The cell growth rate at each tested time point and the number of invasive cells were lower in the 5-Aza-CdR-treated group compared with that in the control group. Flow cytometry revealed that 67.13% of the cells were arrested at the G_0_/G_1_ stage in the 5-Aza-CdR-treated group. The tumorigenic ability of the 5-Aza-CdR-treated group was lower compared with that of the control group. In conclusion, the methylation state of the WWOX gene in HO-8910 cells may be reversed using 5-Aza-CdR, which may also inhibit the growth of these cells.

## Introduction

As epithelial ovarian cancer is difficult to diagnose during the early stages, treatment is often administered later than would be ideal. Epithelial ovarian cancer is the leading cause of gynecological cancer mortalities and poses a serious threat to female health. DNA methylation is a covalent modification of the cytosine residue in a CpG dinucleotide. It is a natural means of controlling gene transcription and does not require any changes to the DNA sequence. DNA methylation is important in the occurrence and development of epithelial ovarian cancer (1,2). The WWOX gene is a newly discovered tumor suppressor gene. It was first isolated and identified using shotgun sequencing, by Bednarek *et al* in 2000 (3). Studies have shown that the primary site of WWOX gene transcription is rich in CpG islands (4). Therefore, methylation may be the key mechanism behind defects in expression. Abnormal methylation of CpG islands in the promoter region of the WWOX gene have been shown to be closely associated with the occurrence and development of breast cancer (5,6). The present study aimed to further explore the correlation between the abnormal methylation of the WWOX gene and epithelial ovarian cancer.

## Materials and methods

### Materials

The HO-8910 human ovarian cancer cell line was obtained from the Department of Obstetrics and Gynecology Laboratory of the Affiliated Hospital of Xuzhou Medical College (Xuzhou, China) and the RPMI-1640 medium was purchased from Hyclone (South Logan, UT, USA). Fetal bovine serum was purchased from Hangzhou Sijiqing Biology Engineering Materials Co., Ltd. (Hangzhou, China). 5-Aza-2′-deoxycytidine (5-Aza-CdR) and MTT were produced by Sigma (St Louis, MO, USA) and the Wizard DNA Clean-up System kits were obtained from Promega (Madison, WI, USA). The Taq DNA polymerases systems were obtained from Qiagen (Hilden, Germany). The oligonucleotide primer was synthesized by Shanghai Shenggong Biological Engineering Co., Ltd. (Shanghai, China). The Transwell chamber and WWOX primary antibody were provided by Chemicon (Temecula, CA, USA). BALB/c female nude mice, aged 4–6 weeks, were supplied by the Shanghai Laboratory Animal Center, Chinese Academy of Sciences (Shanghai, China). This study was approved by the ethics committee of Xuzhou Medical College (Jiangsu, China).

### Cell culture

The HO-8910 cell line was maintained in RPMI-1640 medium (including 70 U/ml penicillin and 70 μg/ml streptomycin) supplemented with 10% fetal bovine serum. The cells were subcultured in a humidified atmosphere of 5% CO_2_ at 37°C in an airtight incubator. Logarithm vegetal period cells were added to the fluid, which included 5.0 μmol/l 5-Aza-CdR, to culture for 24 h. The solution was then replaced by fresh culture fluid containing the same concentration of 5-Aza-CdR. Subsequent to being cultured for 3 days, the solution was replaced by a fresh culture medium that did not contain the drug. The cells were allowed to incubate and the experiment was performed 5 days later.

### WWOX gene methylation state detected by methylation-specific PCR (MSP)

The cells were divided into two groups. Protease K-phenol extraction was used to extract the total DNA, and an ultraviolet spectrophotometer was used to determine the quantity and purity of the DNA. Agarose gel electrophoresis was performed to determine the DNA integrity. The ultraviolet spectrophotometer quantitatively adjusted the final concentration of DNA to 0.1 g/l, and the DNA was stored at −20°C. Subsequently, DNA modification, purification and PCR were conducted. The purification steps of the experiment were performed in accordance with the Wizard DNA Clean-up System kit instructions (Promega). PCR was performed using a Qiagen reaction system. The 20-μl reaction system consisted of 2 μl 10X PCR buffer (Shanghai Shenggong Biological Engineering Co., Ltd.), 0.4 μl dNTP, 0.4 μl upstream and downstream primers, 0.1 μl DNA Taq enzyme, 15.7 μl de-ionized water and 1 μl template. The PCR and MSP amplification conditions were 15 min at 95°C; 40 sec at 95°C, 40 sec at 60°C and 40 sec at 70°C, for 35 cycles; and 10 min at 72°C. An agarose gel electrophoresis was performed and a gel imaging system was used for the scanning analysis. The primer sequences for the methylated WWOX gene were forward, 5′-TATGGGCGTCGTTTTTTTAGTT-3′ and reverse, 5′-CAATCTCCGCAATATCGCGACA-3′. The sequences of the unmethylated primers were forward, 5′-TATGGGTGTTGTTTTTTTAHTT-3′ and reverse, 5′-CAATCTCCACAATATCACAACA-3′. The product size was 347 bp.

### WWOX protein expression detected by western blot analysis

The two groups of cells that were in the growth period were placed in 200 μl cell lysis solution (Shanghai Shenggong Biological Engineering Co., Ltd.) for cracking on ice, and the bicinchoninic acid (BCA) method was used to detect the protein concentration. In order to transfer the proteins to a nitrocellulose membrane, sodium dodecyl sulfate-polyacrylamide gel electrophoresis (SDS-PAGE) was performed. The membranes were incubated with with 5% skimmed milk powder for 60 min, prior to the addition of 1:1,000 anti-WWOX antibody (rabbit anti-human) and incubation overnight at 4°C. The cells were reacted with horseradish peroxidase-labeled sheep anti-rabbit antibody as a secondary antibody (1:10,000; Shanghai Shenggong Biological Engineering Co., Ltd.). This reaction took place at room temperature and was allowed to continue for 2 h. Subsequent to adding enhanced chemiluminescence (ECL) agent (Hangzhou Sijiqing Biology Engineering Materials Co., Ltd.), the mixtures were placed in an anechoic chamber for exposure imaging with highly sensitive X-ray film. The gray value of the target and β-actin protein bands were measured and the ratio of the target band to β-actin indicated the relative expression level of the target protein.

### Proliferation of the HO-8910 cell line detected by MTT

Two groups of HO-8910 cells were obtained in the growth period, and then plated into 96-well plates at a density of 1.5×10^4^/well and cultured for 1–6 days. Following this, 20 μl MTT working fluid was added to each well and incubated in CO_2_ at 37°C for 4 h. Dimethyl sulfoxide was added to terminate the reaction and the absorbance value was detected at 490 nm. Cellular growth curves were constructed based on these results.

### Invasion ability of HO-8910 cells detected by an invasion assay in vitro

Using a Transwell chamber cell invasion assay *in vitro*, an invasion membrane synthesized from matrix adhesive (Matrigel, Promega) was placed between the upper and lower areas of the Transwell chamber. A single HO-8910 cell suspension was vaccinated on the invasion membrane at 200 μl per hole, containing ~10^5^ cells, and cultured at 37°C in 5% CO_2_ for 12 h. The cells and matrix adhesive were wiped on the membrane, which was fixed and stained with H&E. The number of transmembrane cells was calculated using a microscope. Each group of cells had three duplicate wells and the experiment was repeated three times.

### Flow cytometry to detect the HO-8910 cell cycle

The cells that were in the growth period were divided into two groups and digested using 0.25% trypsin. Physiological saline was used for conventional washing. The cells were centrifuged and fixed in 70% ethanol overnight prior to the addition of 100 μl phosphate buffer liquid to disperse them into single cell suspensions. The cells were analyzed using flow cytometry.

### In vivo experiment

#### Animal groups

A total of 20 BALB/c 4- to 6-week-old female nude mice were randomly divided into two groups, a control group and a 5-Aza-CdR-treated group, each containing 10 mice. They were maintained at a constant temperature (25–27°C) and humidity (45–50%), with fresh air and high dust-removing sterilizing and pathogen-free conditions. The mice were allowed free access to food and sterilized water.

#### Animal vaccination and treatment

The two groups of cells in the growth period were digested to form single cell suspensions with a density of 4×10^7^/ml. The cells were inoculated into the abdominal cavities of the nude mice at a volume of 0.5 ml each. The nude mice were observed and the date of death was recorded. A necropsy was performed on the abdominal cavity immediately following death. The volume of abdominal fluid was calculated, and the number, weight and maximum diameter of the tumors and the abdominal viscera metastasis were observed.

#### Western blot analysis to detect WWOX protein expression in the tumor tissue

The tumor tissues from the nude mice in the 5-Aza-CdR-treated and control groups were obtained and weighed. The tumors were then ground with lysis buffer to extract the total protein. These were quantified using the BCA method. SDS-PAGE was performed using a 30-μg protein sample from each group to transfer the proteins to a nitrocellulose membrane. The membranes were incubated with 5% skimmed milk powder for 60 min at room temperature, and then 1:1,000 anti-WWOX antibody (rabbit anti-human) was added and the membranes were incubated overnight at 4°C. The mixture was then allowed to react with horseradish peroxidase-labeled sheep anti-rabbit antibody as a secondary antibody (1:10,000). This reaction took place at room temperature for 2 h. Subsequent to adding ECL agent, the mixture was placed in the anechoic chamber for exposure imaging with a highly sensitive X-ray film.

#### Statistical analysis

All data are expressed as the group mean ± standard deviation, and were processed using SPSS 13.0 statistical software (SPSS, Inc., Chicago, IL, USA). The statistical differences between the groups of data were assessed using the Student’s t-test. P<0.05 was considered to indicate a statistically significant difference.

## Results

### WWOX gene methylation in HO-8910 cells

**Figure 1:**
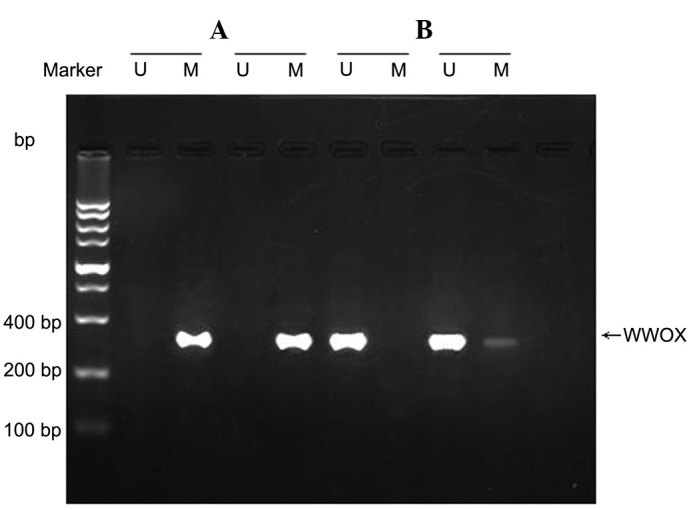
Changes in the methylation state of the WWOX gene in the HO-8910 cell line following treatment with 5-Aza-CdR. (A) Control group and (B) 5-Aza-CdR-treated group. U, unmethylated; M, methylated; 5-Aza-CdR, 5-Aza-2′-deoxycytidine.

The WWOX gene was in a state of methylation in the HO-8910 ovarian cancer cell line. Unmethylated and partially methylated samples were identified following treatment with 5-Aza-CdR (Fig. 1).

### Changes in WWOX expression in 5-Aza-CdR-treated HO-8910 cells

**Figure 2:**
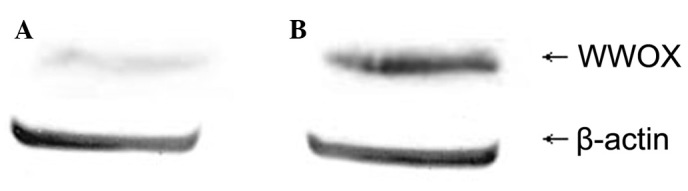
Changes in WWOX expression following treatment with 5-Aza-CdR. (A) Control group. (B) 5-Aza-CdR-treated group. 5-Aza-CdR, 5-Aza-2′-deoxycytidine.

Western blot analysis revealed that the expression level of the WWOX protein in the 5-Aza-CdR-treated group was 0.71±0.023, whereas that of the control group was 0.13±0.012. The difference was identified to be statistically significant (P<0.05; Fig. 2).

### Changes in the proliferation ability of 5-Aza-CdR-treated HO-8910 cells

**Figure 3:**
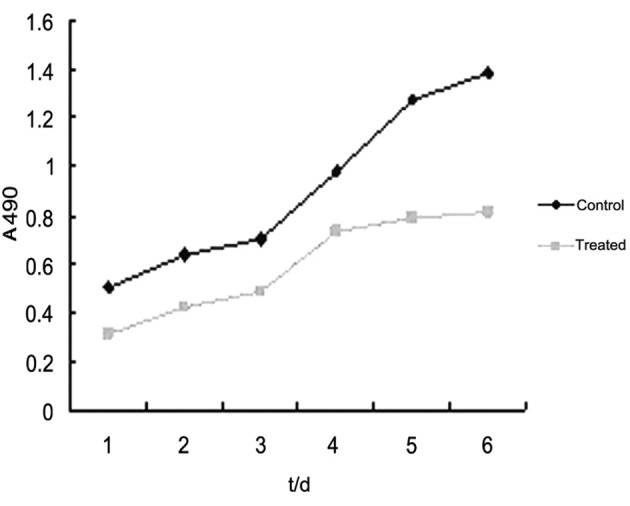
Growth curve of 5-Aza-CdR-treated HO-8910 cells. 5-Aza-CdR, 5-Aza-2′-deoxycytidine; t/d, time/days.

The MTT assay showed that subsequent to being cultured for 1–6 days, the absorbance values of cells in the 5-Aza-CdR-treated group were 0.312±0.013, 0.423±0.015, 0.488±0.022, 0.734±0.031, 0.791±0.019 and 0.814±0.018. The growth rate at each time point was significantly lower than that of the control group at the corresponding time (P<0.05; Fig. 3).

### Changes in the invasion ability of 5-Aza-CdR-treated HO-8910 cells

The *in vitro* invasion assay showed that the number of transmembrane cells in the 5-Aza-CdR-treated group was 92.2±4.7, while 172.1±5.2 transmembrane cells were observed in the control group. This difference was identified to be statistically significant (P<0.05, data not shown).

### Changes in the cell cycle of 5-Aza-CdR-treated HO-8910 cells

Flow cytometry revealed that the cells in the 5-Aza-CdR-treated group apparently arrested in the G_0_/G_1_ phase, and that the number of cells in the S phase was significantly lower than that in the control group (P<0.05; Table I).

### Changes in the tumorigenic ability of 5-Aza-CdR-treated HO-8910 cells in nude mice

The necropsy revealed that transplanted tumors were present in the abdominal cavities of all the nude mice. The tumor formation rate was 100% in the treated and control groups (P>0.05). Each of the nude mice in the control group contained bloody ascites in the abdominal cavity, while two nude mice in the 5-Aza-CdR-treated group showed no significant bloody ascites. The two groups of nude mice had tumor nodules of various sizes scattered in numerous parts of the peritoneum, epiploon, diaphragm, bowel and the surface of the mesentery. Metastasis was observed in the liver, but not in the heart, kidneys, lungs or uterine appendages in the two groups. A comparison between the control and 5-Aza-CdR-treated group revealed significant differences in the survival time, number of metastases, metastatic foci weight, maximum tumor diameter and abdominal volume (P<0.05; Table II).

**Figure 4:**
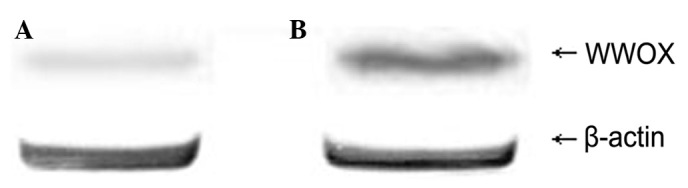
Changes in the expression of WWOX protein in two groups of nude mice. (B) Control group. (B) 5-Aza-CdR-treated group. 5-Aza-CdR, 5-Aza-2′-deoxycytidine.

Western blot analysis showed the expression level of WWOX protein in the tumor issue of the nude mice in the experimental group to be 0.65±0.031, while that of the control group was 0.25±0.047. The difference was statistically significant (P<0.05; Fig. 4).

## Discussion

DNA methylation is the only known natural chemical modification of DNA in mammals. It is one of the main and most studied factors in mammalian epigenetics. In DNA methylation, S-adenosyl-L-methionine supplies the methyl group, which is transferred to the fifth carbon atom of cytosine to generate 5-methylcytosine under the action of the methyltransferase. This may affect gene expression without causing any changes to the gene sequence. The site of DNA methylation is usually located on the CpG island in the promoter region of a gene (7). DNA hypomethylation may promote the expression of tumor suppressor genes, while DNA hypermethylation may decrease or stop the expression of tumor suppressor genes and cause the tumor suppressor genes to lose function. This may result in unrestricted cell growth and ultimately lead to tumorigenesis (8–10).

Epigenetic changes are different from genetic alterations in several ways. One of these variations is that epigenetic changes are able to be reversed. Therefore, the normal regulation of cells is restored by recovering the expression of unmutated genes to achieve the purpose of the treatment. Normal human cell genes are not controlled by CpG island methylation. Therefore, the inhibition of methylation does not affect the expression of genes in normal cells. Genes in static states due to methylation are sensitive to DNA methylation inhibitors and to the abnormal expression of corresponding HLA molecules, allowing gene therapy to recover the activity (11–16). At present, the primary drug for reversing DNA methylation is 5-Aza-CdR. The methyltransferase-specific inhibitor 5-Aza-CdR is a pyrimidine analog whose mechanism is to combine with DNA during DNA replication, form covalent complexes with DNMT1, inhibit the enzyme’s methyl transfer activity, produce low-methyl annihilator chains and reduce the hypermethylation of the gene promoter region to recover gene activity.

The present study revealed that the WWOX gene was in a state of hypermethylation in the HO-8910 ovarian cancer cell line. The cells were in a demethylated state following treatment with 5-Aza-CdR. Due to demethylation, WWOX gene expression increased. A cell growth curve experiment showed a decrease in the growth rate of the HO-8910 cells that were treated with 5-Aza-CdR, suggesting that 5-Aza-CdR may inactivate the WWOX gene through aberrant recovery of methylation. The WWOX gene tumor suppressor function was recovered, which inhibited further growth of the HO-8910 cells. *In vitro* invasion ability was a significant characteristic that was used to measure tumor metastatic potential. A chamber invasion *in vitro* experiment showed that the ability of the HO-8910 cells that were treated with 5-Aza-CdR to invade other tissues decreased markedly, suggesting that the invasion and cancer cell movement abilities were reduced. Flow cytometry showed that the HO-8910 cells were arrested in the S phase following treatment with 5-Aza-CdR. The number of cells in the G_2_/M phases decreased, indicating that the antitumor effect of the WWOX gene may work by affecting the cell cycle following demethylation. In the present study, the HO-8910 cells that were treated with 5-Aza-CdR were also inoculated into the abdominal cavity of nude mice, and the effects of WWOX demethylation on the behavior of the HO-8910 cells were studied further. It was identified that the growth rate of the demethylated HO-8910 cells in the nude mice was significantly slower than that of the control group. Tumorigenicity decreased significantly and the expression levels of the WWOX gene increased in the experimental group. The WWOX gene was observed to be associated with the occurrence and development of ovarian cancer; normal expression of the WWOX gene suppresses the occurrence of ovarian cancer.

In the present study, the *in vivo* and *in vitro* experiments revealed that aberrant methylation of the WWOX gene was the main reason for the reduction in gene transcription and expression, which may be closely associated with the occurrence and development of ovarian cancer. These results also indicated that the tumor suppressor activity was recovered by demethylation of the WWOX gene, which may provide new experimental evidence for the diagnosis and treatment of ovarian cancer.

## Figures and Tables

**Table I:** Changes to the 5-Aza-CdR-treated HO-8910 cellular cycle.

Group	G_0_/G_1_	S	G_2_/M
5Aza-CdR-treated	67.13±0.26	19.56±1.36	13.45±0.38
Control	21.52±0.37	47.09±1.03	31.06±0.61

Values are percentages. Data are presented as the mean ± SD. 5-Aza-CdR, 5-Aza-2′-deoxycytidine.

**Table II:** Comparison of survival time, number of metastases, tumor weight, maximum diameter and abdominal volume between the two groups of nude mice.

Group	No.	Survival time (days)	No. of metastases	Tumor weight (g)	Maximum diameter (mm)	Abdominal volume (ml)
5-Aza-CdR-treated	10	58.3±3.6	33±5	4.73±0.52	4.9±0.7	3.75±0.62
Control	10	37.4±3.1	58±6	9.02±0.17	9.2±0.4	7.53±0.22

Data are presented as the mean ± SD. 5-Aza-CdR, 5-Aza-2′-deoxycytidine.
